# Transcriptomic Analysis of Zebrafish TDP-43 Transgenic Lines

**DOI:** 10.3389/fnmol.2018.00463

**Published:** 2018-12-13

**Authors:** Alexandra Lissouba, Meijiang Liao, Edor Kabashi, Pierre Drapeau

**Affiliations:** ^1^Department of Pathology and Cell Biology and Research Center of the University of Montréal Hospital Center, University of Montreal, Montréal, QC, Canada; ^2^UMR CNRS 1127, UPMC INSERM U 1127, CNRS UMR 7225, Institut du Cerveau et de la Moelle épinière, Sorbonne Université Paris VI, Paris, France; ^3^Institut Imagine, UMR INSERM 1163, Hospital Necker-Enfants, Université Paris Descartes, Paris, France

**Keywords:** amyotrophic lateral sclerosis, ALS, zebrafish, TDP-43, TARDBP

## Abstract

Amyotrophic lateral sclerosis (ALS) is a late-onset progressive neurodegenerative disorder that affects both upper and lower motor neurons, leading to muscle atrophy with spasticity and eventual death in 3–5 years after the disease onset. More than 50 mutations linked to ALS have been found in the gene *TARDBP*, encoding the protein TDP-43 that is the predominant component of neuronal inclusions in ALS. TDP-43 is an RNA binding protein with glycine-rich domains that binds to more than 6,000 RNAs in the human brain. However, ALS-related mutations do not appear to affect the function of these genes, indicating that a toxic gain-of-function may occur. We generated transgenic zebrafish lines expressing human TDP-43, either the wild-type form or the ALS-causative G348C mutation identified in a subset of ALS patients, with the transgene expression driven by an inducible heat shock promoter in order to bypass a potential early mortality. The expression of the mutant but not the wild-type human TDP-43 in zebrafish embryos induced a reduction of the locomotor activity in response to touch compared to controls and moderate axonopathy of the motor neurons of the spinal cord, with premature branching of the main axonal branch, recapitulating previous results obtained by mRNA injections. We used these lines to investigate transcriptomic changes due to the presence of mutant TDP-43 using RNA sequencing and have found 159 genes that are differentially expressed compared to control, with 67 genes up-regulated and 92 genes down-regulated. These transcriptomic changes are in line with recent transcriptomic data obtained in mouse models, indicating that these zebrafish transgenic lines are adequate to further study TDP-43-related ALS.

## Introduction

Amyotrophic lateral sclerosis (ALS) is a devastating motor neuron disorder characterized by the loss of both upper motor neurons, located in the motor cortex and lower motor neurons, located in the anterior horn of the spinal cord and in the brainstem. The disease onset is around 50–60 years of age and is first recognized as muscle weakness, atrophy and eventual death due to respiratory failure, with a median survival of 2–3 years. ALS is part of a clinical continuum with fronto-temporal dementia (FTD), with overlapping genes and clinical features between the two diseases. Most ALS cases appear to be sporadic in nature (sporadic ALS-sALS) and around 10% of patients having a familial history of the disease (familial ALS-fALS) (Chio et al., 2013; Swinnen and Robberecht, 2014; Hardiman et al., 2017). Several genes have been implicated in ALS including *TARDBP*, coding for the protein TDP-43 (Taylor et al., 2016; Therrien et al., 2016).

TDP-43 is a predominantly nuclear protein, but was first linked to ALS and FTD as the main protein found in the ubiquitin-positive cytoplasmic inclusions in the neurons, including motor neurons, of ALS and of FTD patients, with a clearance of its usual nuclear localization (Arai et al., 2006; Neumann et al., 2006). Following this discovery, mutations in *TARDBP* were identified in around 5% of fALS cases, 1% of sALS, cases and 1% of FTD cases (Kabashi et al., 2008; Sreedharan et al., 2008; Caroppo et al., 2016). TDP-43 has several physiological functions mainly in regulating RNAs, as it is involved in most steps of RNA metabolism, including transcription, splicing, transport and stability (Gao et al., 2018). TDP-43 is known to bind to at least 6,000 mRNAs in the murine brain and to modify the expression level of at least 600 mRNAs (Polymenidou et al., 2011), and as such it was hypothesized that mutant TDP-43 may induce differentially expressed genes and isoforms, which may be part of the pathogenicity of ALS.

Several animal models of ALS based on mutations of TDP-43 have been generated, from mouse models to yeast, including zebrafish (Patten et al., 2014; Van Damme et al., 2017), but how ALS-causing mutations of TDP-43 induce the pathophysiological changes seen in ALS patients is still poorly understood. The TDP-43 transgenic mouse models highly overexpress the mutant human TDP-43 protein compared to the endogenous Tdp-43 and result in a very aggressive early onset phenotype with premature lethality (Wegorzewska et al., 2009; Stallings et al., 2010; Xu et al., 2010, 2011). On the other hand, the mice overexpressing an amount of mutant TDP-43 closer to the physiological level display only minor motor deficiencies and have a phenotype that resembles more closely an FTD ones than an ALS one (Swarup et al., 2011; Arnold et al., 2013). Additionally, in most cases, overexpression of the wild-type TDP-43 protein leads to similar defects as the mutated forms, thus preventing the discrimination between the overexpression of a transgene in general and the overexpression of mutant TDP-43 in particular. Recently, a new Tdp-43 mouse model was generated using CRISPR/Cas9 technology to knock-in an endogenous *Tardbp* mutation similar to the human Q331K mutation (White et al., 2018). While these mice presented with motor and cognitive defects, the phenotype did not evolve to paralysis or death after 24 months, thus leading again to a phenotype closer to FTD than ALS.

The overexpression of human mutant TDP-43 but not of wild-type TDP-43 at low doses in zebrafish embryos by mRNA overexpression has been shown to result in reduced locomotion and aberrant motor neuron axons, with abnormal synaptic transmission (Kabashi et al., 2010, 2011; Laird et al., 2010; Vaccaro et al., 2012, 2013; Armstrong and Drapeau, 2013; Patten et al., 2017). However, these models express only transiently the human protein and the high level of expression may vary with the quality of the mRNA injections, adding variability to the models. To palliate this issue, we aimed to generate stable transgenic TDP-43 zebrafish lines expressing human TDP-43 with ALS-causing mutations in order to study the disease using zebrafish in a more accurate fashion. Thus, we have generated two transgenic zebrafish lines expressing either human wild-type TDP-43 or human TDP-43 bearing the G348C mutation in ALS and characterized the locomotor activity and the axonal defects of these lines. We also identified differentially expressed genes due to the presence of mutant TDP-43. Using these lines, we performed an unbiased transcriptomic analysis comparing whole transcriptome from zebrafish expressing mutant TDP-43 to control and we identified candidate genes that may be involved in TDP-43 pathogenicity.

## Material and Methods

### Ethics Statement

This study was approved by the Canadian Council for Animal Care and conducted at the Research Center of the University of Montreal Hospital Center (CRCHUM).

### Fish Husbandry

Wild-type zebrafish (*Danio rerio*) were maintained at 28.5°C and kept under a 12/12 h light/dark cycles at the animal facility of the Centre Hospitalier de Montréal research center, Montréal, Canada. They were bred according to standard procedure (Westerfield, 1995) and staged as previously described (Kimmel et al., 1995).

### Generation of the Transgenic Lines and Heat Shock Procedure

The pCS2-TDP43-Myc and pCS2-TDP43-G348C-Myc plasmids were generously shared by Dr. Guy Rouleau (McGill University). They respectively contain the human wild type *TARDBP* cDNA and the human *TARDBP* cDNA bearing the G1176T change encoding for the G348C mutation. Six sequences coding for the myc tag were added to the 3′ end of the sequences. The Tol2Kit was used to generate the two pDest-Tol2CG2-hsp70-TDP43-Myc and pDest-Tol2CG2-hsp70-TDP43-Myc vectors. 25 ng/μl of these vectors were injected in one-cell stage zebrafish embryos with 25 ng/μl of the Tol2 transposase mRNA with 0.05% Fast Green (Sigma). The injections were done using a Picospritzer III pressure ejector (General Valve, Fairfield, NJ, USA). Approximately 300 embryos were injected for each line and < 40% expressed eGFP in the heart. These embryos were selected and raised to adulthood as the F0 founders. For the TDP-43^WT^ line, 25 F0 fish reached sexual maturity and 11 of them transmitted the transgene to their progeny when outcrossed with wild type AB zebrafish, as identified by eGFP expression in the heart. The percentage of transgene transmission for these F0 founders varied from 0.69 to 33.54%. We selected the two F0 fish with the highest transgene transmission rate (28.44 and 33.54%) to raise the F1 generations. The adult F1 TDP-43^WT^ fish were then outcrossed to identify the rate of transgene transmission and estimate the copy number of the transgene. The transmission rate of one of the TDP-43^WT^ lines followed normal mendelian ratios for a single insertion, with 50% of the progeny inheriting the transgene and we decided to only work with this line. 32 TDP-43^G348C^ F0 fish reached adulthood and only 7 of them transmitted the transgene to their progeny, at a rate of 0.78–29.42%. Due to the low percentages, only the two F0 fish transmitting with the highest frequency per line (17.95 and 29.42%) were selected to raise their progeny until adulthood, to create the two independent lines A-TDP-43^G348C^ and B-TDP-43^G348C^. The adult F1 fish were then outcrossed to identify the rate of transgene transmission and both TDP-43^G348C^ lines displayed mendelian ratios of transgene transmission, with around 50% of transgene transmission.

For the heat shock procedure, 20 hpf embryos were transferred to glass tubes with system water. The tubes were then placed in a pre-heated incubator at 38.5°C for 60 min at a 60 RPM agitation. Embryos were then put back at 28.5°C and dechorionated at 25 hpf.

### Western Blot

Western blots were performed as previously described (Gan-Or et al., 2016). Briefly, about 30 embryos for each condition were lysed in ice-cold RIPA buffer (150 mM NaCl, 50 mM Tris pH 7.5, 1% Triton X-100, 0.1% SDS, 1% Na deoxycholate, 0.1% protease inhibitor), maintained on ice and homogenized with a hand-held pestle. The lysates were centrifuged for 10 min at 10,000 rpm at 4°C and the supernatants were collected. Protein concentration was established using Bio-Rad DC Protein Assay and 60 μg of proteins were loaded in 2X Lämmli buffer after boiling the samples for 5 min at 95°C. TGX Stain-Free FastCast acrylamide Kit (Bio-Rad) was used to make 10% Tris-glycine. After electrophoresis, the gels were activated by exposure to UV for 1 min, and transferred to either regular or low-fluorescence PVDF membrane (Bio-Rad). After transfer, total proteins on membranes were detected by UV. Membranes were then blocked for 1 h using 5% fat free milk in PBST. The primary antibody (anti-myc, 1:5,000) was incubated overnight at 4°C in 5% fat free milk in PBST. After washing 3 × 5 min in PBST, the membrane was incubated with an anti-mouse IgG, HRP conjugated (1:5,000, Jackson Immuno) in 5% fat free milk in PBST for 1 h at room temperature, and then washed 3 × 5 min. Membranes were exposed to Clarity enhanced chemiluminescence (ECL, Bio-Rad) for 5 min at room temperature and visualized using a ChemiDoc MP (Bio-Rad). Detection and quantification of band intensities was conducted using Image Lab 5.2 software (Bio-Rad).

### Immunohistochemistry

Whole-mount immunohistochemistry was done like previously described (Dzieciolowska et al., 2017). Briefly, 2 dpf embryos were fixed in 4% paraformaldehyde overnight at 4°C. Following fixation, the embryos were rinsed several times over the course of an hour with phosphate buffered saline and incubated in 1 mg/ml collagenase in PBS for 40 min. The collagenase was washed off over 1 h with PBS and the embryos were then incubated with Triton X-100 (PBST) for 30 min. Following this step, the embryos were incubated in fresh block solution prepared from PBS containing goat serum, bovine serum albumin, dimethyl sulfide (DMSO) and Triton X-100, for 1 h at room temperature and then treated with a solution containing a primary antibody against pre-synaptic synaptotagmin 1 (znp-1) (1:100; Molecular Probes) overnight at 4°C. Samples were then washed in PBST and incubated in block solution containing a secondary antibody (Alexa Fluor 488, 1:1000; Invitrogen) for 6 h at 4°C. Before imaging, larvae were transferred to a solution containing 70% glycerol and mounted the following day on a slide. The embryos were visualized using a Quorum Technologies spinning disk confocal microscope with a CSU10B (Yokogawa) spinning head mounted on an Olympus BX61W1 fluorescence microscope and connected to a Hamamatsu ORCA-ER camera. Images were acquired using Volocity software (Improvision) and analyzed using ImageJ (NIH).

### Touch-Evoked Escape Response

Locomotor activity was assessed by lightly touching the tail of the zebrafish embryos with a light forceps. The embryos were placed at the center of a 150 mm diameter petri dish filled with system water that was kept at 28.5°C by a heating plate. Swimming was recorded from above at 30 Hz (Grasshopper 2 camera, Point Gray Research) and swim duration, swim distance and maximum swim velocity were quantified off line using the Manual Tracking plug-in for ImageJ (NIH).

### RNA-Sequencing

Total RNA from around 100 RNAlater-fixed embryos (Ambion) was extracted using RNAsolv reagent (Omega Biotek) following the manufacturer's standard protocol. RNA extraction was made using between 5.19 and 9.75 × 105 cells by RNAsolv reagent manufacturer's protocol (Omega Biotek). Absence of contamination with chemicals was assessed by nanodrop using 260/280 and 260/230 ratios. Quantification of total RNA was made by nanodrop and 0.1–1.44 g of total RNA was used for sequencing. Quality of total RNA was assessed with the BioAnalyzer Nano (Agilent).

Library preparation was done with the Truseq RNA (Illumina). 18 PCR cycles was required to amplify cDNA libraries. Libraries were quantified by nanodrop and BioAnalyzer. All librairies were diluted to 10 nM and normalized with the Miseq SR50 v2. Libraries were pooled to equimolar concentration and multiplexed by six samples per lane. Sequencing was performed with the Illumina Hiseq2000 using the SBS Reagent Kit v3 (100 cycles, paired-end) with 1.6 nM of the pooled library. Cluster density was targeted at around 800 k clusters/mm^2^. Around 70 million reads were generated for each sample. Library preparation and sequencing was done at the Institute for Research in Immunology and Cancer's Platform (University of Montreal). About 95% of high quality reads were mapped onto GRCz10 version of the zebrafish genome (gene annotation from Ensembl version 87) using STAR version 2.5.1b.

Differential gene expression analysis was assessed by DeSeq package using R software. Differential gene expression was filtered on a False Discovery Rate (or adjusted *p*-value) < 0.05.

### qRT-PCR

Quantitative RT-PCR (qRT-PCR) primers for each gene were designed using the Universal Probe Library tool from Roche. Total RNA was extracted from FACS-purified NSCs using RNAsolv reagent (Omega Biotek) and chloroform followed by isopropanol precipitation. Reverse transcription was performed from 1 μg of total RNA using the superscript VILO reverse transcription mix (Invitrogen). Quantitative PCR was performed on 2 μL of 1:10-diluted cDNA using SYBR Green I master (Roche) on a LightCycler 80 thermocycler. *polr2d* gene (ENSDART00000108718) was used as a reference gene for ddCt quantification.

## Results

### Generation of Transgenic TDP-43 Zebrafish

**Figure 1:**
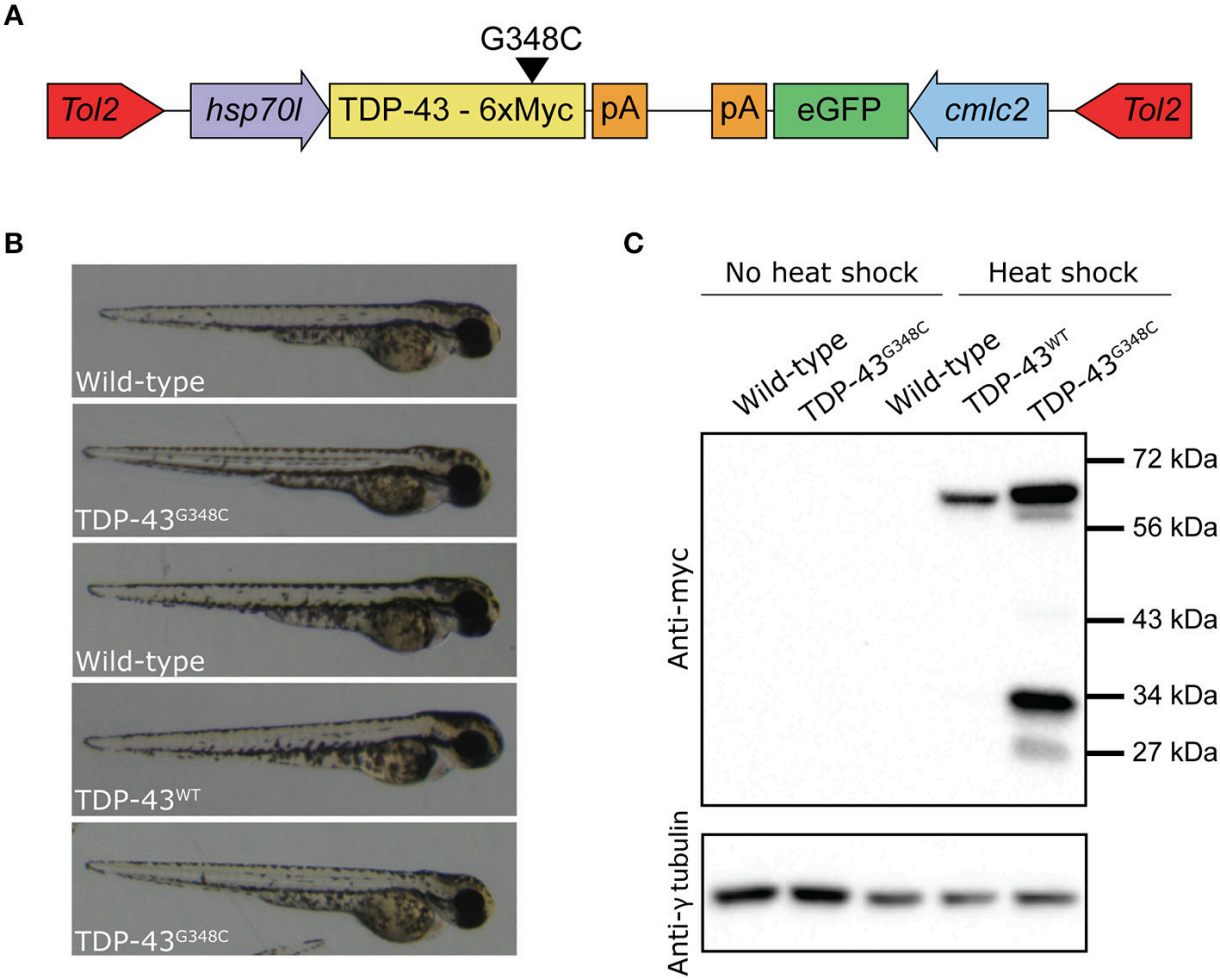
Generation of zebrafish transgenic lines expressing wild type and mutant human TDP-43. **(A)** The transgene constructs consist of the human wild type (TDP-43^WT^) or the G348C mutant TDP-43 (TDP-43^G348C^) cDNA fused with 6 myc-tag under the control of the hsp70-like promoter (*hsp70l*). The constructs also contained eGFP driven by the cardiac myosin light chain (*cmlc2*) promoter. **(B)** Representative picture of the embryos used for all experiments. **(C)** Western blot showing expression of the human transgene in 2 days post-fertilization (dpf) embryos following a 1 h heat shock at 38.5°C using an anti-myc antibody.

In order to study the effects of mutant TDP-43, we have generated transgenic zebrafish lines expressing stably either human wild-type TDP-43 (TDP-43^WT^) or bearing the G348C mutations found in ALS patients from a French-Canadian population (TDP-43^G348C^) (Kabashi et al., 2008). The transgenic lines were generated using the human TDP-43 cDNA to which 6 myc tags were fused to the C-terminus of the protein. Since previous work showed that the presence of mutant TDP-43 in the early stages of development of the zebrafish lead to adverse effects (Kabashi et al., 2010, 2011; Laird et al., 2010; Vaccaro et al., 2012, 2013; Armstrong and Drapeau, 2013; Patten et al., 2017), we decided to use an inducible promoter, the zebrafish heat shock protein 70 like promoter (*hsp70l*), to temporally control the expression of the transgene. The transgene constructs also contained eGFP driven by the cardiac myosin light chain 2 (*cmlc2*) promoter used as a reporter gene in order to easily identify transgenic embryos with green hearts (Figure 1A).

The constructs were injected in 1–4 cell stage embryos (F0) and the fish were raised until adulthood. The adult F0 fish were outcrossed with wild-type fish and their progeny was screened for the presence of fluorescent hearts, to select the ones having integrated the transgenes in their germline. We selected the two F0 fish with the highest transmission rate for both lines to raise the F1. These F1 fish were outcrossed with wild-type to estimate the transgene copy number by the transgene transmission rate. While both TDP-43^G348C^ lines had an ~50% transmission rate of the transgene, only one of the TDP-43^WT^ line transmitted the transgene following mendelian ratios and only this line was kept for further experiments. We selected the TDP-43G348C line (B) that had the closest level of expression to the TDP-43WT line (Supplementary Figure S1). The F2 offspring were raised to adulthood and used to establish the lines for the following experiments.

In order to express the transgene, a 60 min heat shock at 38.5°C (10°C above the temperature at which they are raised) is done when the embryos are 20 h post-fertilization (hpf). Only normal looking embryos are selected at 48 hpf for experiments (Figure 1B). To establish the expression of the transgenes by Western blot, we used an anti-myc antibody. No transgenic protein was detectable before the heat shock, thus validating the integrity of the *hsp70l* promoter. However, upon heat shock the TDP-43^WT^ and TDP-43^G348C^ transgenes were detectable at around 60 kDa, which corresponds to the molecular weight of the TDP-43 protein with the linker and the 6 myc tags (Figure 1C). Interestingly, we observed the TDP43^WT^ expression level was systematically lower than that of TDP-43^G348C^. Moreover, several bands were also observed in the TDP-43^G348C^ line, indicating the presence of C-terminus TDP-43 fragments, consistent with what is seen in human patients and in mouse studies (Stallings et al., 2010; Swarup et al., 2011; Xu et al., 2011; Gao et al., 2018) (Figure 1C).

### Locomotor Defects Induced by the TDP-43^G348C^ Transgene

**Figure 2:**
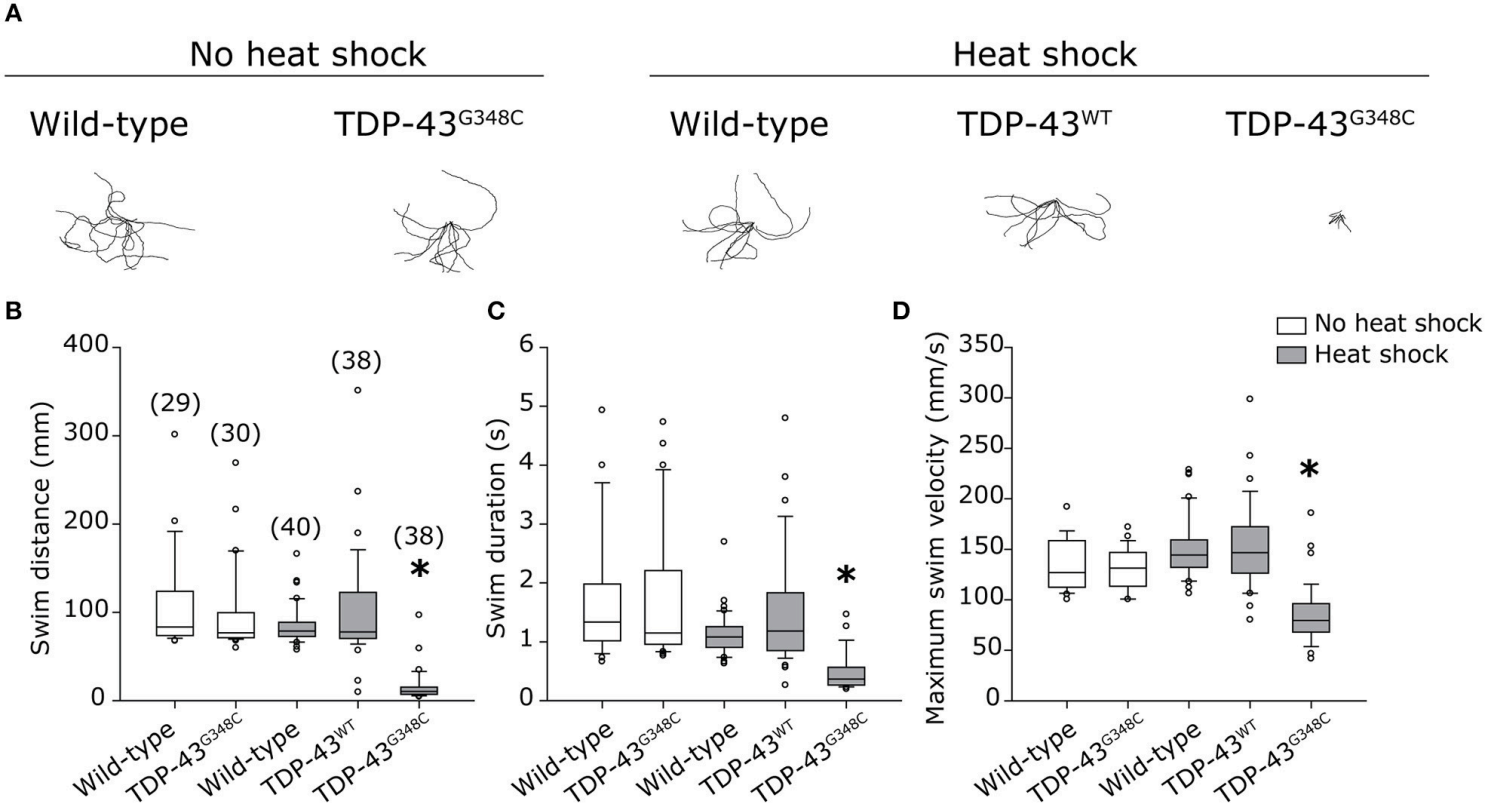
Zebrafish embryos expressing TDP-43^G348C^ display an abnormal touch-evoked locomotion at 2 dpf. **(A)** Overlay of 10 representative swim traces obtained after lightly touching the tail of 2 dpf embryos with a blunt forceps. Embryos expressing TDP-43^G348C^ exhibited a significantly reduced swim distance **(B)**, swim duration **(C)** and maximum swim velocity **(D)**, compared to embryos expressing TDP-43^WT^ or not expressing any transgene. All experiments were done in triplicate; the total number of embryos used is indicated in brackets. Kruskal-Wallis one-way analysis on variance on ranks was performed and pairwise multiple comparison procedures were done according to Dunn's method. *p-value < 0.05.

We then verified if the stable expression of TDP-43^G348C^ upon heat shock was associated with locomotor defects, as it was observed in previous studies by transient expression. We elicited the touch-evoked response by lightly touching the tail of 48 hpf embryos with a blunt forceps. The swim distance, swim duration and the maximum swim velocity were analyzed using the swim traces obtained by tracking the embryos following the touch (Figure 2A). While heat shock of wild type embryos or expressing TDP-43^WT^ had no influence on locomotion compared to wild type, the expression of TDP-43^G348C^ led to a strong reduction of the swim distance, the swim duration and the maximum swim velocity compared to controls (Figure 2B), indicating that the motor neuron axonal defects had a strong impact on the physiology as seen by the severe negative influence on the locomotion of the TDP-43^G348C^ embryos, as was previously reported (Kabashi et al., 2010, 2011; Laird et al., 2010; Vaccaro et al., 2012, 2013; Armstrong and Drapeau, 2013; Patten et al., 2017).

### Human Mutant TDP-43 Induces Motor Neuron Axonopathy

**Figure 3:**
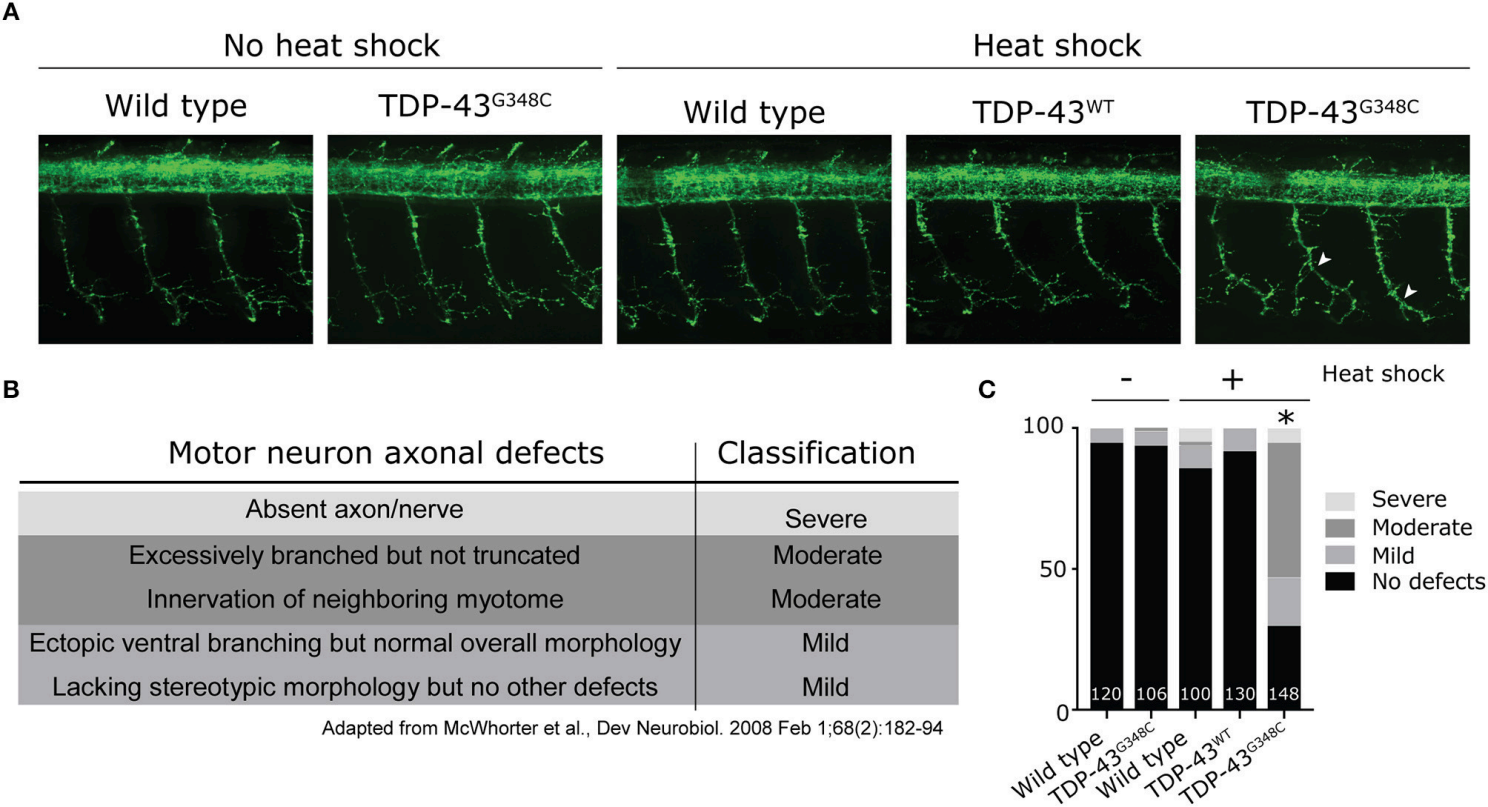
Abnormal development of motor neuron axons in zebrafish expressing TDP-43^G348C^. **(A)** Immunohistochemistry against znp-1 in 2 dpf whole-mount embryos. The 3-to-4 somites spanning the anus are shown with the axons of the motor neuron expanding down from the spinal cord. White arrow heads indicate abnormal premature branching of the main axonal branch and absence of secondary branching in transgenic embryos expressing TDP-43^G348C^, compared to embryos expressing TDP-43^WT^ or not expressing any transgene. **(B)** Classification criteria used for the quantification of defects. **(C)** Quantification of axonal defects based on the classification found in **(B)**. The total number of axons analyzed is indicated; The three axons spanning the anus were analyzed in at least 30 embryos over the course of three experiments. Chi-square analysis was performed between the different conditions, *p-value < 0.05.

As locomotor defects were observed, we investigated whether they reflected axonal motor neuron defects by immunohistochemistry using an antibody against the pre-synaptic marker znp-1. When TDP-43^G348C^ was expressed, the majority of motor neuron axons exhibited several defects, including overbranching of the main axonal branch, absence of secondary branching or abnormal innervation of nearby myotome (Figure 3A). The axonal defects were classified as mainly moderate (Figure 3C) using a classification scale based on the visual interpretation of the axonal appearance, adapted from McWhorter et al (Figure 3B) (McWhorter et al., 2008). The overexpression of the TDP-43^WT^ transgene, or the heat shock of wild-type embryos did not induce specific defects, indicating that it was the specifically the presence of mutant TDP-43 rather than the heat shock or the presence of a foreign gene that was responsible for the defects obtained with TDP-43^G348C^. This is consistent with previous zebrafish models overexpressing mutant TDP-43 (Kabashi et al., 2010, 2011; Laird et al., 2010; Vaccaro et al., 2012, 2013; Armstrong and Drapeau, 2013; Patten et al., 2017). Altogether, these data validate the relevance of our stable expressing transgenic zebrafish lines since they display a clinically-relevant pathogenicity associated with the G348C mutation.

### Transcriptomic Analysis of TDP-43^G348C^ Embryos and qRT-PCR Validation

Given that TDP-43 is an RNA-binding protein involved in RNA metabolism (Gao et al., 2018), we next sought to investigate whether transcriptomic modifications were caused by the expression of TDP-43^G348C^. While TDP-43 is involved in mRNA splicing, it is also involved in mRNA stability and the depletion of Tdp-43 causes around 600 mRNAs to have altered expression levels in mice (Polymenidou et al., 2011). Thus, we used RNA-sequencing to look at differentially regulated genes due to TDP-43^G348C^ expression. Since the human TDP-43^WT^ line following heat shock presented no obvious phenotype compared to wild-type zebrafish with heat shock, and since the expression level of human TDP-43^WT^ was lowered than human TDP-43^G348C^ with an equal heat shock, we hypothesized that using human TDP-43^WT^ as a control may give us differentially expressed genes based on the expression level of the transgenes rather than solely based on the presence of the mutation. We thus decided to use the wild type zebrafish heat shock condition as a control in order to compensate for the effect of the heat shock.

**Figure 4:**
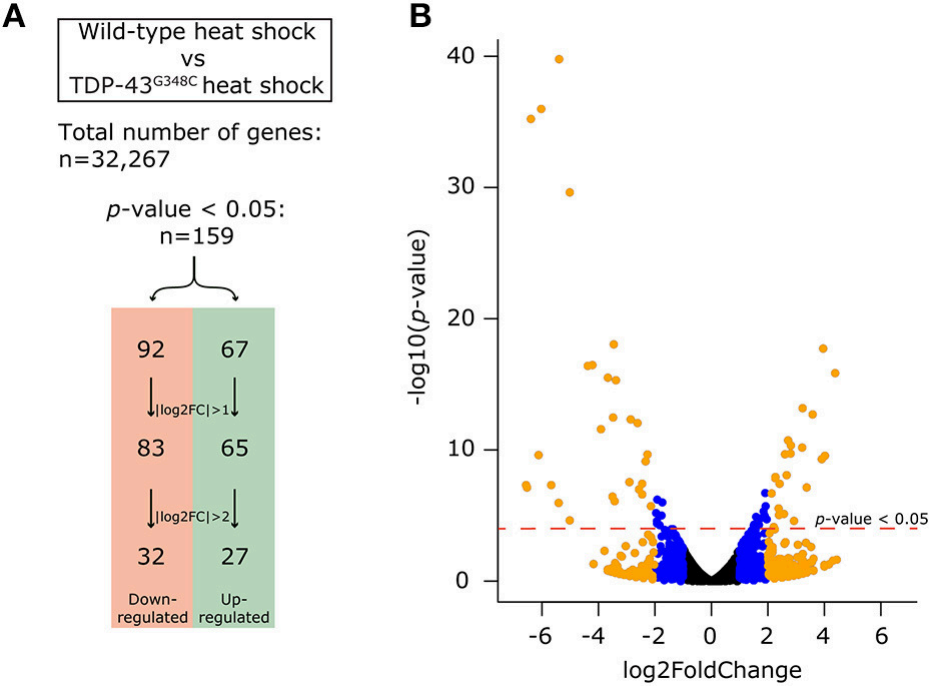
Differential expression analysis obtained by RNA-sequencing. **(A)** In order to compensate for the effect of the heat shock, we compared the expression of genes between the wild-type heat shock and the TDP-43^G348C^ heat shock conditions. Out of the 32,267 genes identified, 159 genes (67 up-regulated and 92 down-regulated) had a significant differential expression (*p*-value < 0.05). This number could be reduced by using a more stringent cut-off of either a |log2FC| > 1 (148 genes, 65 up-regulated and 83 down-regulated) or |log2FC| > 2 (59 genes, 27 up-regulated and 32 down-regulated). **(B)** Volcano plot showing all of the 32,267 genes. The dotted red line indicates the level of significance with *p*-value < 0.05. The blue dots show the genes with a |log2FC| comprised between 1 and 2. The orange dots show the genes with |log2FC| > 2.

More than 70 million reads were sequenced and analyzed for either TDP-43^G348C^ heat shock or wild type heat shock and more than 95% were mapped to the GRCz10 version of the zebrafish genome (gene annotation from Ensembl version 87). We used DESeq to analyze the differentially expressed genes amongst the 32,267 genes analyzed between the two conditions. Filtering on a *p*-value < 0.05 we found 159 differentially expressed genes, with 67 up-regulated and 92 down-regulated. In order to have a more stringent cut-off, we used a |log2FC| greater than one, corresponding to a minimal 2-fold up-regulation or down-regulation. This cut-off decreased the number of differentially expressed genes to 148 (65 up-regulated and 83 down-regulated). An even more stringent cut-off of |log2FC| greater than two, corresponding to a minimal 4-fold up- or down- regulation, led to only 59 genes that were differentially regulated, with 27 up-regulated and 32 down-regulated (Figure 4A). A volcano plot representing these data is shown in Figure 4B with data in, blue, orange or black for cut-offs of |log2FC| greater than two or one or for *p* < 0.05, respectively. The list of significantly differentially expressed genes can be found in the Supplementary Table S1.

These results show that the expression of a G348C mutant TDP-43 is associated with broad changes in the transcriptome. While no common pathways could be found between the 159 genes with *p* < 0.05, some of these changes can be associated with potential pathophysiological mechanisms of ALS and TDP-43 mutations. Thus, the human orthologs of some of the differentially expressed genes have been linked to several neuromuscular disorders, including ALS and muscular dystrophy, such as *CLCN2, ELOVL4, ELOVL5, PEA15, LBH, PAMR1*, and *MSTO1* (references in Table 1). Other genes can be linked to pathways that are perturbed in ALS, such as calcium signaling (*icn2, smdt1a, pvalb4, pvalb9* and *adcy1b*), or mitochondria and oxidative stress (*msto1, smdt1a*, and *mt2*). Also of interest, some of the genes identified are unknown, such as *ponzr3, ponzr4*, or *ftr86*, which may indicate new pathogenic mechanisms of ALS (references in Table 1).

**Table 1:** Genes of interest from the RNA-sequencing data.

**Gene name**	**Log2 FC**	**Gene description**	**Comments**	**References**
*clcn2b*	1.45	Chloride channel, voltage-sensitive 2b	Disease (*CLCN2*: leukoencephalopathy with ataxia; familial hyperaldosteronism type 2); ion channels	Depienne et al., 2013; Scholl et al., 2018
*elovl7b*	2.27	ELOVL fatty acid elongase 7b	Disease (*ELOVL4*: SCA-34; *ELOVL5*: SCA-38); ER-membrane proteins	Cadieux-Dion et al., 2014; Di Gregorio et al., 2014; Ozaki et al., 2015
*pvalb9*	−1.08	Parvalbumin 9	Disease (*PVALB*: ALS); calcium signaling	Nihei et al., 1993
*pvalb4*	1.13	Parvalbumin 4		
*fgfbp2b*	1	Fibroblast growth factor binding protein 2b	Disease (*FGFBP1*: ALS); *FGFBP1*: NMJ; *FGFBP3*: anxiety	Yamanaka et al., 2011; Valdez et al., 2014; Taetzsch et al., 2017
*smdt1a*	1.89	Single-pass membrane protein with aspartate-rich tail 1a	Disease (*SMDT1*: Limb-girdle muscular dystrophy); mitochondria; calcium signaling	Sancak et al., 2013; Monies et al., 2017
*pea15*	1.56	Proliferation and apoptosis adaptor protein 15	Disease (Parkinson-like disorder)	Perruolo et al., 2016
*lbh*	3.96	Limb bud and heart development	Disease (Alzheimer's disease)	Yamaguchi-Kabata et al., 2018
*pamr1*	2.72	Peptidase domain containing associated with muscle regeneration 1	Disease (Duchenne muscular dystrophy)	Nakayama et al., 2004
*msto1*	1.28	Misato 1, mitochondrial distribution and morphology regulator	Disease (mitochondrial myopathy and ataxia; cerebellar atrophy with pigmentary retinopathy); mitochondria	Gal et al., 2017; Nasca et al., 2017; Iwama et al., 2018
*adcy1b*	2.03	Adenylate cyclase 1b	Calcium signaling (neurone specific, calmodulin-sensitive)	Sethna et al., 2017
*icn2*	−1.74	Ictacalcin 2	Calcium signaling	Kraemer et al., 2008
*mt2*	−1.27	Metallothionein 2	Oxidative stress	Shimazu et al., 2013
*ftr86*	1.41	FinTRIM family, member 86	Unknown, possible antiviral activity	Luo et al., 2017
*ponzr4*	−2.56	Plac8 onzin related protein 4	Unknown	

*Genes of interest amongst the differentially expressed genes, upon expression of human mutant TDP-43. The full list of differentially expressed genes can be found in Supplementary Table S1*.

**Figure 5:**
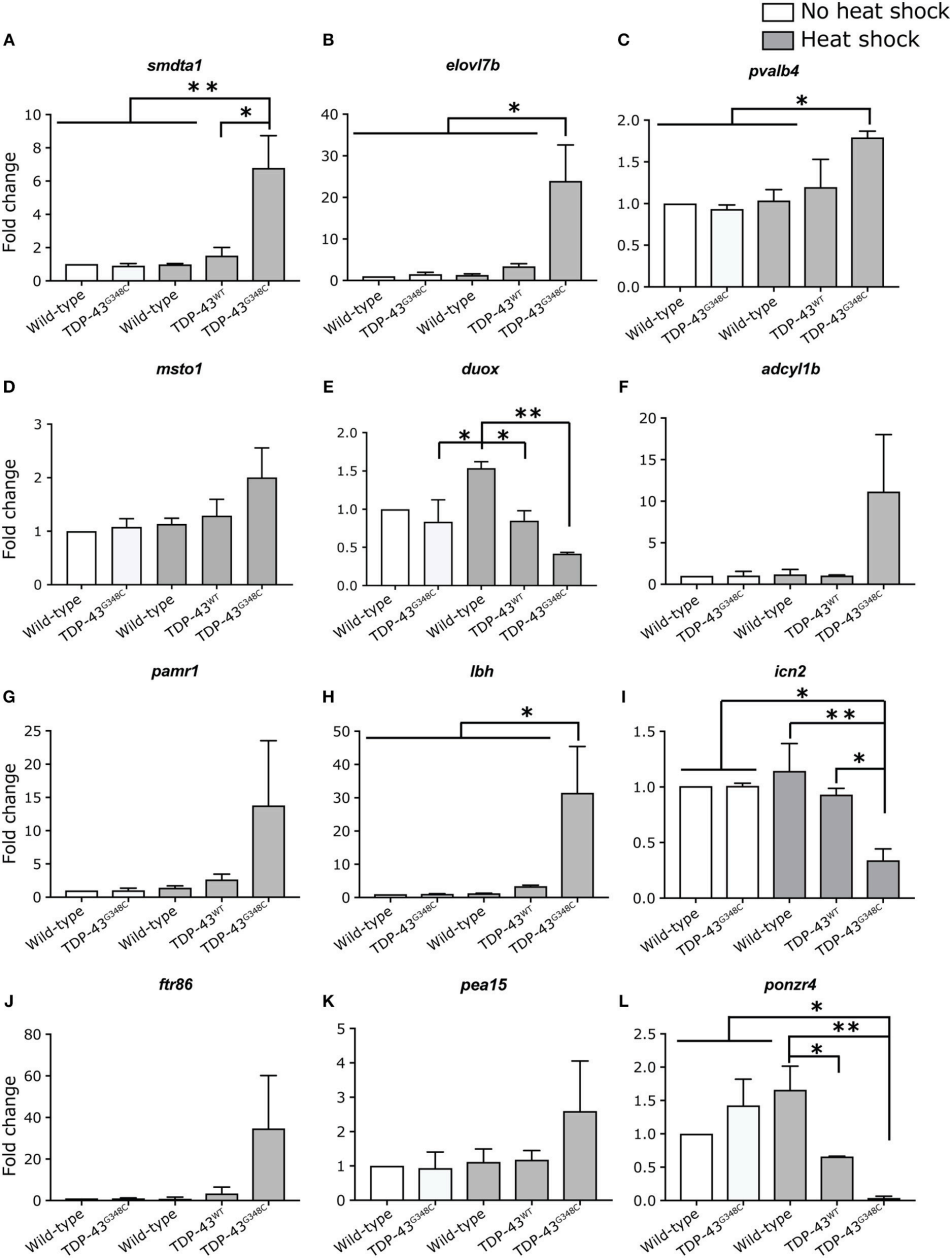
Validation of differentially expressed genes by RT-qPCR. **(A–L)** Expression level of 12 selected genes was tested in duplicate by RT-qPCR in all of the conditions. One-way ANOVA with Tukey's multiple comparisons test was performed. *p-value < 0.05, **p-value < 0.01 and error bars are standard deviation.

In order to validate these results, we selected 12 of the differentially expressed genes to measure their expression levels by qRT-PCR in all of our conditions, including TDP-43^WT^ heat shock (Figures 5A–L). All mRNA expression levels positively correlated with the RNA-sequencing data, even though significance was not always reached (Figures 5D–G,J,K), probably due to variability in the level of mRNA expression (Figures 5D,F,I,J).

## Discussion

In this report, we have generated inducible transgenic zebrafish lines expressing human TDP-43, either wild type or bearing the ALS-causing mutation G348C. We have characterized the phenotype induced by the expression of the mutant TDP-43^G348C^ and have used this line to study transcriptomic variations induced by ALS-causing mutant TDP-43.

Overexpressing mutant TDP-43 but not wild-type at 21 hpf, during a critical period for motor neuron development (Myers et al., 1986), induced a reduction of the locomotor activity in response to touch with reduced swim distance, time and velocity compared to controls. These swimming defects were correlated with moderate axonopathy of the motor neurons of the spinal cord, with premature branching of the main axonal branch and branching defects in the secondary branches. These results recapitulate the phenotype obtained by overexpression of mutant TDP-43 mRNA by our group and others (Kabashi et al., 2008, 2010; Laird et al., 2010; Vaccaro et al., 2012, 2013; Armstrong and Drapeau, 2013; Patten et al., 2017), thus validating our transgenic lines as genetic models of TDP-43-related ALS that can be used to better understand the pathophysiology of TDP-43 pathogenicity in ALS.

Interestingly, a stronger transgene expression was systematically reached for TDP-43^G348C^ compared to TDP-43^WT^. It was shown to be the case in mouse models as well, including in the transgenic mouse model expressing the same G348C mutation from Swarup and colleagues (Igaz et al., 2011; Swarup et al., 2011) and may be explained by a higher stability of the mutant protein. It is unlikely to be the result of a deficiency in TDP-43 autoregulation due to the G348C mutations, since we generated our transgenic lines using cDNA lacking the TDP-43 3′UTR necessary for its autoregulation (Ayala et al., 2011). However, the fact that we observed multiple bands by western blot when TDP-43^G348C^ was expressed may indicate a dysfunction in its normal degradation, thus impacting the turnover of the protein. An additional possibility inherent to the Tol2 system used to generate these transgenic lines is that the TDP-43^G348C^ line may have integrated concatemers of the transgene at a single insertion point, thus leading to a higher expression level of the transgene compared to the TDP-43^WT^ line, while still following mendelian ratios.

Since TDP-43 affects the level of many mRNAs (Polymenidou et al., 2011), we used our transgenic lines to identify differentially expressed genes at the time when we observed the TDP-43^G348C^ mutant induced phenotype. We found 159 genes misregulated compared to heat shocked wild type embryos, the majority with 2- to 4-fold differences. However, since we did not use TDP-43^WT^ expressing embryos as a control, we cannot exclude that some of the transcriptomic changes identified may be due to the presence of a transgene and not specifically to the G348C TDP-43 mutant. However, we validated a subset of these genes by qRT-PCR in both transgenic lines and found concordant results, with up- or down-regulation of the selected genes specific to the expression of TDP-43^G348C^ and not TDP-43^WT^. Testing of genetic interactions between these potential new ALS hits and TDP-43 will allow us to functionally validate these results.

White and colleagues investigated transcriptomic differences in their Tdp-43 knock-in mice (White et al., 2018). In the lumbar region of 5-months-old mice, only 31 genes were found to be differentially regulated. However, when repeating this experiment using the frontal cortices of these mice, 404 genes were differentially regulated and this number increased to 1,219 when the experiment was done in 20 months-old mice (White et al., 2018). Even though our experimental paradigms are different, with an induced early onset vs. a slow disease course, it is interesting to note that 16 differentially expressed genes or their orthologs are in common between our two datasets. Additionally, 12 of these common genes were found when comparing our dataset to their aged mouse frontal cortex dataset. While this may be due to chance, it could also indicate that some of these transcriptomic changes may be the consequences of the phenotype, rather than its cause (Supplementary Table S1). Our results presented here indicate that these zebrafish transgenic lines are promising tools to better understand TDP-43 pathophysiology and will complement other existing models.

## Data Availability Statement

The raw data supporting the conclusions of this manuscript will be made available by the authors, without undue reservation, to any qualified researcher.

## Author Contributions

EK and ML generated the transgenic lines. AL designed and interpreted the experiments. AL and PD wrote the manuscript. All authors reviewed the manuscript.

### Conflict of interest statement

The authors declare that the research was conducted in the absence of any commercial or financial relationships that could be construed as a potential conflict of interest.
