# Supplementary material for: Transcriptomic Analysis of Zebrafish TDP-43 Transgenic Lines
Source: Front Mol Neurosci. 2018 Dec 13;11:463. doi: 10.3389/fnmol.2018.00463 (PMC6301209; doi:10.3389/fnmol.2018.00463)
Supplement: Supplementary file 1 [file Data_Sheet_1.docx]

Supplementary Material

Transcriptomic Analysis of Zebrafish TDP-43 Transgenic Lines

Alexandra Lissouba, Meijiang Liao, Edor Kabashi, Pierre Drapeau*

*** Correspondence:** Dr. Pierre Drapeau: p.drapeau@umontreal.ca

# Supplementary Figures and Tables

## Supplementary Figure S1


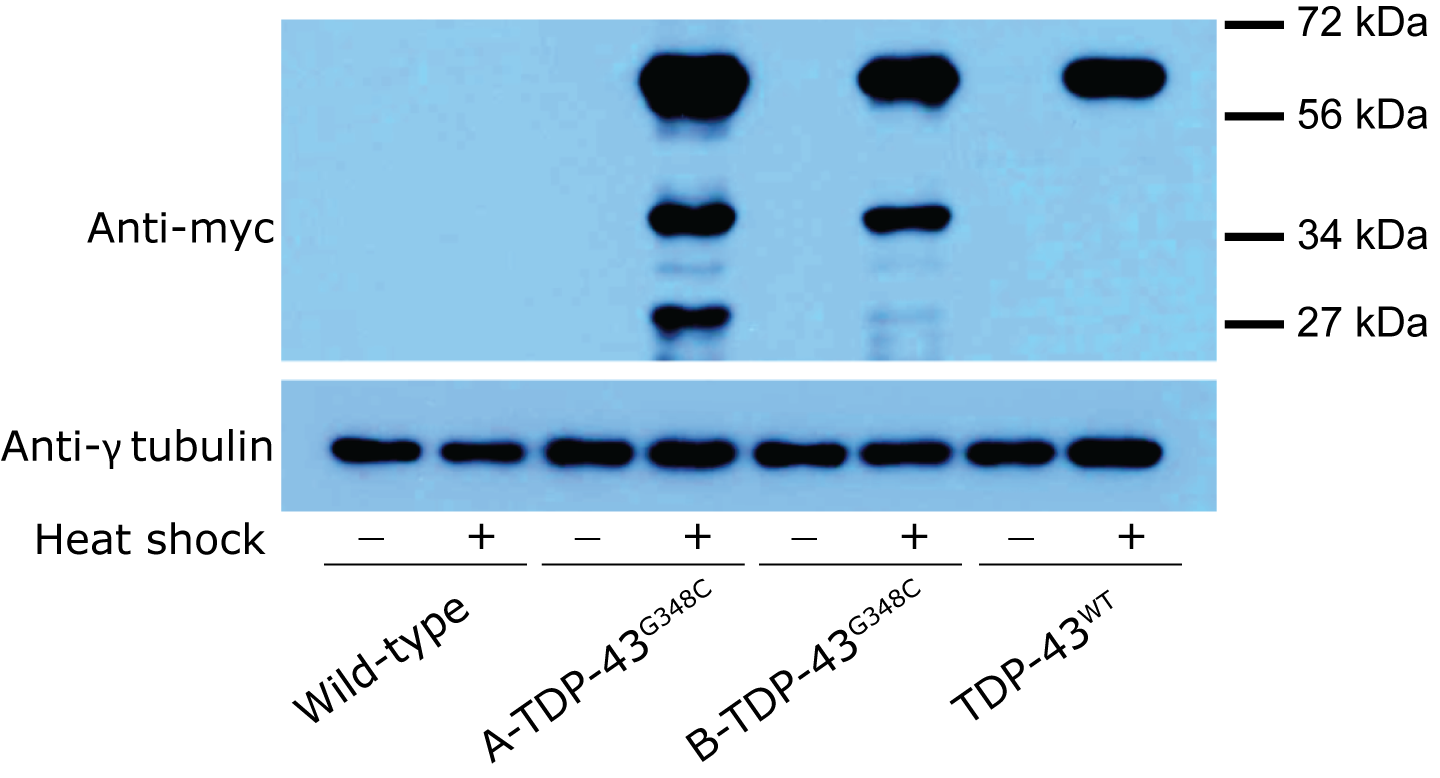


**Figure S1.** Western blot showing the expression of the human transgenes at 2 days post fertilization without (-) or with (+) a one hour heat shock at 38.5°C. The conditions shown are wild-type embryos, embryos from one independent TDP-43^G348C^ line (A-TDP-43^G348C^), a second independent TDP-43^G348C^ line (B-TDP-43^G348C^) and from the TDP-43^WT^ line.

## Supplementary Table S1

**Table S1**. Differentially expressed genes upon expression of human mutant TDP-43**.** We found 159 differentially expressed genes in embryos expressing human mutant TDP-43 compared to heat-shock wild-type embryos by filtering with a *p*-value < 0.05. 67 genes are up-regulated (green cells) and 92 genes are down-regulated (red cells). The log2 fold change (log2FC), the p value and the base mean values from the DESeq analysis have been provided for each gene. Genes highlighted in blue are the ones with homologs that are also misregulated in the study from White and colleagues (White et al., 2018).
